# Comment on “The Combined Extract of *Zingiber officinale* and *Zea mays* (Purple Color) Improves Neuropathy, Oxidative Stress, and Axon Density in Streptozotocin Induced Diabetic Rats”

**DOI:** 10.1155/2015/593734

**Published:** 2015-05-05

**Authors:** Rômulo Dias Novaes, Reggiani Vilela Gonçalves

**Affiliations:** ^1^Department of Structural Biology, Federal University of Alfenas, 37130-000 Alfenas, MG, Brazil; ^2^Department of Animal Biology, Federal University of Viçosa, 36570-000 Viçosa, MG, Brazil

In a recent study, Wattanathorn et al. identified that the combined extract of purple waxy corn and ginger was effective in improving functional and structural changes in STZ-diabetic rats submitted to chronic constriction of the sciatic nerve, an effect that is potentially mediated by the antioxidant properties of these combined extracts. Although the results have been quite interesting, we would like to present a complementary conceptual perspective to improve the interpretation of the findings considering the disease model investigated. In the natural course of nontreated diabetes mellitus, diabetic neuropathy represents a chronic metabolic neuropathic disorder secondary to accumulated molecular and microvascular damage that impairs nerve structure and function [1, 2]. Fundamentally, diabetic neuropathy consists of a nontraumatic condition with prolonged time-course in which functional impairment overcomes the nerve structural damage, especially in the early stages of neuropathy [1–3]. Thus, even when applied to diabetic animals, the model of nerve constriction represents a traditional model of traumatic neuropathy and neuropathic pain [4, 5], which manifests a completely different pathophysiologic mechanism compared to diabetic neuropathy. In this trauma model, nerve ligation causes variable degrees of injury, which is difficult to measure directly and can range from neuropraxia (less severe functional nerve lesion) to neurotmesis (extensive and complex morphofunctional nerve lesion) [4, 5]. Furthermore, the injured nerve fibers cause chromatolysis and distal Wallerian degeneration, processes that are associated with intense focal inflammation and fibrosis, which limits axonal reparation [4, 5]. All of these structural changes are typical of traumatic lesions, but less frequent in diabetic neuropathy [1–3]. From this conceptual basis, the current interpretation of the findings reflects a construct bias since the results described cannot be used as an empirical basis to explain the improvements in diabetic neuropathy. Thus, a proper interpretation of the findings should consider the effect of treatments on healing/repair of traumatic nerve injury in diabetic animals, which recognizably presents an impaired healing process [1, 3]. When reviewing animal models of diabetic neuropathy, Klusáková and Dubový [5] indicated that rats with partial sciatic nerve ligation present minor pathogenesis of diabetic neuropathy, which is hardly representative of the human condition. This argument is reinforced by the short time course for neuropathy evolution, which showed a significant impairment of sciatic function index 24 hours after nerve ligation. This finding indicates a typical acute effect of mechanical nerve injuries [4, 5], which is not consistent with the chronic and progressive clinical course of diabetic neuropathy [1–3]. Although the relevance of the results is evident in traumatic nerve injury, it is wise to discuss the limitations of the model used and the interpretation of the findings. Whereas diabetic neuropathy is a condition of difficult clinical management that demands high cost to health care, we are looking forward to further studies by Wattanathorn et al. investigating whether the extracts tested can also bring similar benefits in specific and robust models, such as genetic or genetically modified models [3, 5, 6] of diabetic neuropathy.
